# Cost-utility of NeuroSAFE-guided RARP Versus Standard RARP in Men with Localised Prostate Cancer in the UK

**DOI:** 10.1016/j.euros.2026.08.007

**Published:** 2026-09-09

**Authors:** Jiunn Wang, Ricardo Almeida–Magana, Eoin Dinneen, Shengning Pan, Baptiste Leurent, Nicholas Roberts, Chris Brew–Graves, Norman R. Williams, Aiman Haider, Greg Shaw, Elena Pizzo

**Affiliations:** a Department of Primary Care and Population Health, University College London, London, UK; b Division of Surgery and Interventional Science, University College London, London, UK; c Department of Urology, University College London Hospitals NHS Foundation Trust, London, UK; d Department of Statistical Science, University College London, London, UK; e Centre for Medical Imaging, University College London, London, UK; f Department of Histopathology, University College London Hospitals NHS Foundation Trust, London, UK; g School of Public Health, Imperial College London, White City Campus, London, UK; h Business School, Imperial College London, South Kensington Campus, London, UK

**Keywords:** Cost-utility analysis, NeuroSAFE, Prostate cancer

## Abstract

**Background:**

Robotic radical prostatectomy (RARP) is a standard first-line curative treatment for localised prostate cancer but carries risks of erectile dysfunction and urinary incontinence. The NeuroSAFE technique provides pathological assessment to optimise nerve-sparing whilst avoiding positive surgical margins.

**Objective:**

This study evaluated the cost-effectiveness of NeuroSAFE-guided RARP compared with standard RARP.

**Design, setting and participants:**

A within-trial economic evaluation with a 12-month time horizon was conducted alongside NeuroSAFE-PROOF, a single-blinded, multi-centre, randomised controlled trial. Participants were patients with a diagnosis of non-metastatic prostate cancer deemed suitable to undergo RARP, good erectile function without medical erectile function assistance, and no previous prostate cancer treatment. No age limits were applied.

**Intervention:**

The intervention is the NeuroSAFE-guided RARP.

**Outcome measurements and statistical analysis:**

Costs were assessed from NHS and societal perspectives using patient-level data. Quality-adjusted life years (QALYs) were measured using EQ-5D-5L. Incremental cost-effectiveness ratios (ICERs) and net monetary benefits were estimated. Uncertainty was captured via bootstrapping, missing data were addressed through multiple imputation, and Monte Carlo simulations were undertaken to assess robustness.

**Results and limitations:**

Among 407 randomised patients (NeuroSAFE: 204; standard RARP: 203), NeuroSAFE increased mean total cost (by £827 and £1,000 from NHS and societal perspectives, respectively) and QALYs (by 0.03). ICERs were £26,195/QALY (NHS) and £31,666/QALY (societal). Cost-effectiveness probabilities were 53% and 56% (NHS) and 48% and 56% (societal) at the lower and upper UK willingness-to-pay thresholds. Limitations include substantial uncertainty around estimates, missing data, and potential recall bias from retrospectively collected data. Findings reflect short-term, within-trial estimates and should be interpreted accordingly.

**Conclusions and Patient summary:**

NeuroSAFE-guided RARP increased costs and generated modest QALY gains on average. ICERs were within the upper UK threshold, although substantial uncertainty remained. These findings reflect 12-month within-trial economic outcomes and should not be interpreted as evidence of long-term cost-effectiveness.

## Take Home Message

Magnetic resonance imaging based quantification of Gleason pattern 4/5 enables accurate long-term prediction of metastatic recurrence after radical prostatectomy. Although peripheral zone tumors showed worse outcomes in unadjusted analyses, zonal origin was not an independent predictor after multivariable adjustment and should not be used for routine risk stratification without further validation.

ADVANCING PRACTICE **What does this study add?**

This is the first cost-utility analysis of the NeuroSAFE technique conducted within a large multicentre randomised controlled trial in the UK, and it uses robust and detailed data on participants’ sociodemographic characteristics, resource utilisation, and utility measures at multiple time points.

Analyses were conducted from multiple economic perspectives. Sensitivity analyses supported the direction of the findings, although uncertainty remained.

**Clinical Relevance**

Using data from the NeuroSAFE-PROOF trial, NeuroSAFE-guided RARP may improve quality-adjusted survival at 12 months compared with standard RARP, but at an additional cost. The estimated cost per QALY falls within commonly used UK willingness-to-pay thresholds, although substantial uncertainty remains around the estimates. These findings may support the use of NeuroSAFE in selected patients, but longer-term data are needed to establish its cost effectiveness.

**Patient Summary**

In this study, the NeuroSAFE technique was associated with small improvements in quality-adjusted life years on average over 12 mo compared with standard surgery. Although clinical trial results suggest improvements in urinary continence and erectile function, the overall quality-of-life gains were modest and should be interpreted in the context of short-term follow-up and incomplete data. These findings apply to men with good baseline erectile function included in the trial.

## 1 Introduction

When diagnosed at a localised stage, prostate cancer (PC) is amenable to cure by radical treatments such as robot-assisted radical prostatectomy (RARP). However, erectile dysfunction and urinary incontinence are common side effects of this procedure. With more than 8000 patients undergoing RARP each year in England alone [1], the patient and societal burden of these side effects is substantial.

Preserving the prostatic neurovascular bundles, or nerve-sparing (NS), during RARP can reduce the incidence of these functional side effects but may increase the risk of leaving residual cancer behind (positive margins), especially when extra-prostatic extension (EPE) is present [2]. In standard practice, surgeons select patients for NS RARP based on clinical, biopsy, and imaging information to predict the risk of EPE. However, this leads to many patients not receiving NS when, in retrospect, the tumour was confined to the prostate [3].

First described in Germany [4], the NeuroSAFE technique (intraoperative frozen section examination of the neurovascular structure adjacent prostate margin) provides real-time pathological consult to guide optimal NS while avoiding positive margins. We recently published the 12-mo results of the first randomised controlled trial (RCT) comparing the use of NeuroSAFE against standard practice, showing a statistical and clinically significant improvement in erectile function and urinary continence in favour of the NeuroSAFE arm [5]. However, no publications have explored the impact of this procedure in quality of life and cost effectiveness of this procedure.

This paper presents the 12-mo cost-utility analysis of the NeuroSAFE-PROOF trial conducted across five hospitals in the United Kingdom (UK).

## 2 Methods

### 2.1 NeuroSAFE trial design

We have previously published the protocol and primary results of the NeuroSAFE-PROOF Study [5], [6]. Briefly, NeuroSAFE-PROOF was an investigator-led, IDEAL stage 3, multicentre, patient-blinded, parallel RCT conducted across five National Health Service (NHS) centres in the UK comparing NeuroSAFE-guided RARP versus standard RARP. Key inclusion criteria included good baseline erectile function (International Index of Erectile Function [IIEF]-15 sum score ≥22 on the first five items without medical assistance) [7] and no previous PC treatment. Participants were randomised 1:1 to either NeuroSAFE-guided RARP or standard RARP, and were informed of their NS status, but remained unaware of whether the NeuroSAFE technique had been used. Randomisation was performed using a centralised, secure web-based system with allocation concealment. Treatment assignment was not accessible to investigators or recruiting clinicians prior to patient enrolment, ensuring that allocation could not be predicted or influenced.

### 2.2 Intervention description

Standard RARP was performed according to each participating centre’s standard technique.

The NeuroSAFE-guided group underwent initial intrafascial NS dissection on both sides regardless of preoperative recommendations, followed by immediate histopathological examination of the inked posterolateral margins and a full secondary resection performed if significantly positive according to our published protocol.

### 2.3 Data

Data was collected at baseline, and at 3-, 6-, and 12-mo postsurgery follow-up. A full description of the questionnaires used can be found in the protocol paper [5]. Health care utilisation was captured via the Client Service Receipt Inventory (CSRI) questionnaire [8] and health-related quality of life (HRQoL) assessed using the European Quality of Life-5 Dimensions-5 Levels (EQ-5D-5L) [9], [10].

### 2.4 Overview of economic evaluation

Outcomes were measured using quality-adjusted life years (QALYs), consistent with National Institute for Health and Care Excellence (NICE) recommendations [11]. Cost-utility was expressed as the incremental cost per additional QALY gained.

The evaluation adopted both an NHS and Personal Social Services (PSS) and a societal perspective [11]. Health care resource use was assessed using trial data, with unit costs derived from UK standard published sources [12], [13], [14]. Costs were expressed in 2023/24 British pounds and inflated where appropriate [15]. A 12-mo time horizon was employed, corresponding with the trial’s primary follow-up period. Given this short duration, no extrapolation or discounting of costs and outcomes was applied.

### 2.5 Resource use and costs

#### 2.5.1 Costs of intervention and comparator

A micro-costing approach was used to identify resources specifically associated with the NeuroSAFE procedure. Costs common to both surgical approaches (eg, robotic platform use, standard surgical consumables, and preoperative care) were assumed to be identical across groups and were therefore not included in the incremental cost calculation. The incremental cost of NeuroSAFE reflects additional operating theatre time and pathology resources required for intraoperative frozen section analysis; these assumptions on cost were based on results from the feasibility study, NHS tariffs [12], Personal Social Service Research Unit 2023/4 [14], and the British National Formulary 2023/4 [13]. Table 1 reports a detailed breakdown of cost components.

**Table 1:** Costs of NeuroSAFE-guided RARP and standard RARP: mean costs per participant

Activity and resources	Control	Intervention	Additional	RARP surgery cost	NeuroSAFE cost
**Surgery cost**					
General anaesthetic		1 h extra	1 h		£15
Da Vinci robot-assisted consumables tariff	same	same	none	£7076	£7076
Mean time of surgery (under anaesthetic)	2 h 13 min	3 h 16 min	1 h		
Member of staff takes prostate to lab interoperative (courier)	none	10 min	10 min		£5
Member of staff takes prostate to routine NHS pathology at the end of the day	same	same	none		
**NeuroSAFE technique**					
Consultant histopathology		1 h	1 h		£143
Two biomedical laboratory technicians (1 h each)		2 h	2 h		£66
**Additional time in theatre**					
Consultant surgeon		1 h	1 h		£141
Registrar surgeon		1 h	1 h		£72
Consultant anaesthetist		1 h	1 h		£143
Two scrub nurses (band five)		1 h	1 h		£96
Two health care assistants (band three)		1 h	1 h		£72
**Total cost**				**£7076**	**£7829**
**Difference in cost**					**£753**

BNF = British National Formulary; NHS = National Health Service; PSSRU = Personal Social Services Research Unit; RARP = robot-assisted radical prostatectomy.

Source: Our elaboration using trial data and NHS tariffs, PSSRU 2023/24, and BNF 2023/24.

#### 2.5.2 Costs of NHS and PSS services

NHS and PSS service costs per participant were calculated by multiplying recorded service utilisation by published unit costs [12], [13], [14]. These services included general practitioner consultations, specialist consultations, oncologic consultations, and treatment, including any additional or secondary therapies received during follow-up, Accident and Emergency (A&E) attendance, hospital admissions, outpatient appointments, and medication usage. Resource utilisation was collected retrospectively at each visit using an adapted version of the CSRI.

For the societal perspective, additional costs comprised out-of-pocket private health care expenses and productivity losses due to time off work associated with surgery and postoperative appointments, calculated using average daily salary data from the CSRI.

### 2.6 QALY

Generic HRQoL was assessed via the EQ-5D-5L questionnaire [9] administered at baseline and at 3-, 6-, and 12-mo follow-ups. EQ-5D-5L responses were converted into utility scores using UK population–specific valuation weights [16], [17]. Utility scores range from negative values, representing health states worse than death, to 0 (death) and 1 (full health). Participant-specific utility profiles were generated by linear interpolation between utility values recorded at each measurement point. QALYs were computed as the area under each participant’s utility profile from baseline to 12-mo follow-up.

### 2.7 Statistical analysis

We estimated the effect of assignment to NeuroSAFE-guided RARP versus standard RARP (intention-to-treat estimand), reflecting a policy-relevant comparison of adopting the NeuroSAFE technique in clinical practice. Mean differences in costs and QALYs between groups were estimated using separate linear regression models with treatment assignment as the only independent variable. Incremental cost-effectiveness ratios (ICERs) were calculated as the ratio of incremental costs to incremental QALYs. Net monetary benefits (NMBs) were estimated using NICE willingness-to-pay thresholds (£25 000–£35 000 per QALY) [11].

Our sample includes patients who underwent adjuvant radiation therapy, which is no longer standard of care following changes in evidence since the study began. To assess its potential impact, we conducted a sensitivity analysis excluding these patients.

### 2.8 Missing data

Missing costs and QALYs were jointly imputed using multiple imputation by chained equations with predictive mean matching [18], [20]. Age, sociodemographic variables, and clinical variables, including baseline prostate volume, prostate-specific antigen, clinical T stage, Cambridge Prognostic Groups classification, IIEF-5, and International Consultation on Incontinence Questionnaire Short Form, were included as explanatory variables. Fifty imputed datasets were generated, reflecting the approximately 50% proportion of incomplete cases [19], [20], and results were pooled according to Rubin’s rules [21].

Data were assumed to be missing at random (MAR). Uncertainty was assessed using nonparametric bootstrapping with 1000 replicates performed within each imputed dataset. For robustness, we further conducted complete-case analyses and assessed departure from MAR assumptions using a pattern-mixture model [22].

### 2.9 Probabilistic sensitivity analysis

Probabilistic sensitivity analysis was conducted to evaluate the robustness of our findings. Statistical distributions were assigned to model parameters: γ distributions were used for costs, and β distributions for utilities. Uncertainty was captured using bootstrapping with 1000 replicates per imputed dataset [18]. Cost-effectiveness acceptability curves [23] were generated to illustrate the probability of cost effectiveness across different willingness-to-pay thresholds.

### 2.10 Interaction analysis

A subgroup of participants not recommended for bilateral NS at preoperative planning was found to derive greater clinical benefit from NeuroSAFE [5], [6]. To examine the economic benefit in this subgroup, we conducted additional analyses by regressing NMB on treatment assignment alone (Model 1), and then including a binary subgroup indicator and a treatment-subgroup interaction term (Model 2) as an exploratory analysis.

## 3 Results

A total of 407 participants were randomised to either standard RARP (*n* = 203) or NeuroSAFE-guided RARP (*n* = 204). Baseline assessments were completed by 391 participants, with follow-up EQ-5D data obtained from 299 (3 mo), 286 (6 mo), and 289 (12 mo), and resource use data from 223, 230, and 254 participants at the same time points, respectively.

### 3.1 Costs of NeuroSAFE-guided RARP and standard RARP

The estimated cost of performing standard RARP was £7076 and £7829 for NeuroSAFE-guided RARP (Table 1). The £753 difference was attributable to additional staff costs and longer theatre time.

### 3.2 Costs of health resource use

Supplementary Table 1 reports health resource use costs for the observed data. The main cost drivers were medical devices, hospital outpatient admissions, family and paid carer support, and productivity losses. Costs were broadly similar between arms, except for family and paid carer support, where the NeuroSAFE group incurred consistently higher average spending (NeuroSAFE-guided RARP: £82.83 at 3 mo and £98.42 at 12 mo; standard RARP: £59.38 at 3 mo and £14.24 at 12 mo).

### 3.3 QALY

Baseline EQ-5D-5L utility scores were comparable (NeuroSAFE: 0.89; standard RARP: 0.89). At 3 mo, utilities slightly favoured NeuroSAFE (NeuroSAFE: 0.88; standard RARP: 0.84), with minimal differences at 6 mo (NeuroSAFE: 0.87; standard RARP: 0.88) and 12 mo (NeuroSAFE: 0.87; standard RARP: 0.84). The full trajectory of utility scores is presented in Table 2.

**Table 2:** Trajectories of utility scores, based on observed data

**Group**	**Baseline**		**3 mo**		**6 mo**		**12 mo**	
	***n* (*n/N*)**	**Mean (SD)**	***n* (*n/N*)**	**Mean (SD)**	***n* (*n/N*)**	**Mean (SD)**	***n* (*n/N*)**	**Mean (SD)**
Standard RARP	197 (97%)	0.89 (0.13)	152 (75%)	0.84 (0.20)	147 (72%)	0.88 (0.14)	142 (70%)	0.84 (0.20)
NeuroSAFE-guided RARP	194 (95%)	0.89 (0.12)	147 (72%)	0.88 (0.13)	139 (68%)	0.87 (0.16)	147 (72%)	0.87 (0.19)

RARP = robot-assisted radical prostatectomy; SD = standard deviation.

### 3.4 Cost-utility

From an NHS perspective, NeuroSAFE-guided RARP generated an incremental cost of £827 per participant (95% confidence interval [CI] = £651–£1003) and an increase in QALYs (0.03; CI = –0.01 to 0.21), yielding an ICER of £26,195/QALY. This exceeds the lower NICE threshold (£25 000/QALY) but falls below the upper threshold (£35 000/QALY). NMBs favoured NeuroSAFE at the upper threshold (£22 124 for NeuroSAFE-guided RARP versus £21 846 for standard RARP) but not the lower (£13 387 for NeuroSAFE-guided RARP versus £13 425 for standard RARP; Table 3). From a societal perspective, total average costs were higher for NeuroSAFE (£8947) than RARP (£7947), with an ICER of £31 666/QALY (Table 4).

**Table 3:** Base-case results following multiple imputation: mean costs, QALYs, ICER, and NMB (NHS perspective)

	Standard RARP	NeuroSAFE-guided RARP
Intervention cost	£7076	£7829
Health service cost	£550	£624
Total cost	£7626	£8453
Incremental cost [95% CI]		£827 [£651–£1003]
QALYs	0.84	0.87
Incremental QALYs [95% CI]		0.03 [–0.01 to 0.21]
ICER (extra cost per QALY gained)		£26 195
Net monetary benefit (CE threshold: 25 000)	£13 425	£13 387
Net monetary benefit (CE threshold: 35 000)	£21 846	£22 124
Incremental NMB (CE threshold: 25 000)		–£38
Incremental NMB (CE threshold: 35 000)		£278

CE = cost-effectiveness; CI = confidence interval; ICER = incremental cost-effectiveness ratio; NMB = ‘‘net monetary benefit; NHS = National Health Service; QALYs = quality-adjusted life years; RARP = robot-assisted radical prostatectomy.

Values represent mean estimates from regression analyses pooled across imputed datasets using Rubin’s rules.

Confidence intervals were derived from nonparametric bootstrap using the percentile method.

**Table 4:** Base-case results following multiple imputation: mean costs, QALYs, ICER, and NMBs (societal perspective)

	**Standard RARP**	**NeuroSAFE-guided RARP**
Intervention cost	£7076	£7829
Health service cost	£728	£979
Productivity loss	£142	£138
Total cost	£7947	£8947
Incremental cost [95% CI]		£1000 [£697–£1338]
QALYs	0.84	0.87
Incremental QALYs [95% CI]		0.03 [–0.01 to 0.21]
ICER (extra cost per QALY gained)		£31 666
Net monetary benefit (CE threshold: 25 000)	£13 105	£12 894
Net monetary benefit (CE threshold: 35 000)	£21 525	£21 630
Incremental NMB (CE threshold: 25 000)		–£211
Incremental NMB (CE threshold: 35 000)		£105

CE = cost-effectiveness; CI = confidence interval; ICER = incremental cost-effectiveness ratio; NMB = net monetary benefit; QALYs = quality-adjusted life years; RARP = robot-assisted radical prostatectomy.

Values represent mean estimates from regression analyses pooled across imputed datasets using Rubin’s rules.

Confidence intervals were derived from nonparametric bootstrap using the percentile method.

Complete-case analyses and sensitivity analyses excluding patients who received adjuvant radiation therapy showed results consistent in direction with the imputed base-case analysis (Supplementary Tables 2 and 3). Sensitivity analyses exploring departure from the MAR assumption also showed similar directional results for incremental costs and QALYs, although with greater uncertainty around cost effectiveness (Supplementary Table 4).

### 3.5 Probabilistic sensitivity analysis and cost-effectiveness acceptability curve

Probabilistic sensitivity analyses showed similar trends to the base-case results, although substantial uncertainty remained. From an NHS perspective, NeuroSAFE was on average £827 more expensive (95% CI = £574–£1088), with slight differences in QALYs (0.03; 95% CI = –0.01 to 0.21). NMBs were lower for NeuroSAFE at the lower threshold (£13 387 vs £13 422 for NeuroSAFE-guided RARP and standard RARP, respectively) but higher at the upper threshold (£22 123 vs £21 841 for NeuroSAFE-guided RARP and standard RARP, respectively; Supplementary Table 5). From a societal perspective, the mean incremental cost was £1001 per participant with an ICER of £31 600/QALY, which was within the upper NICE threshold (Supplementary Table 6).

**Fig. 1:**
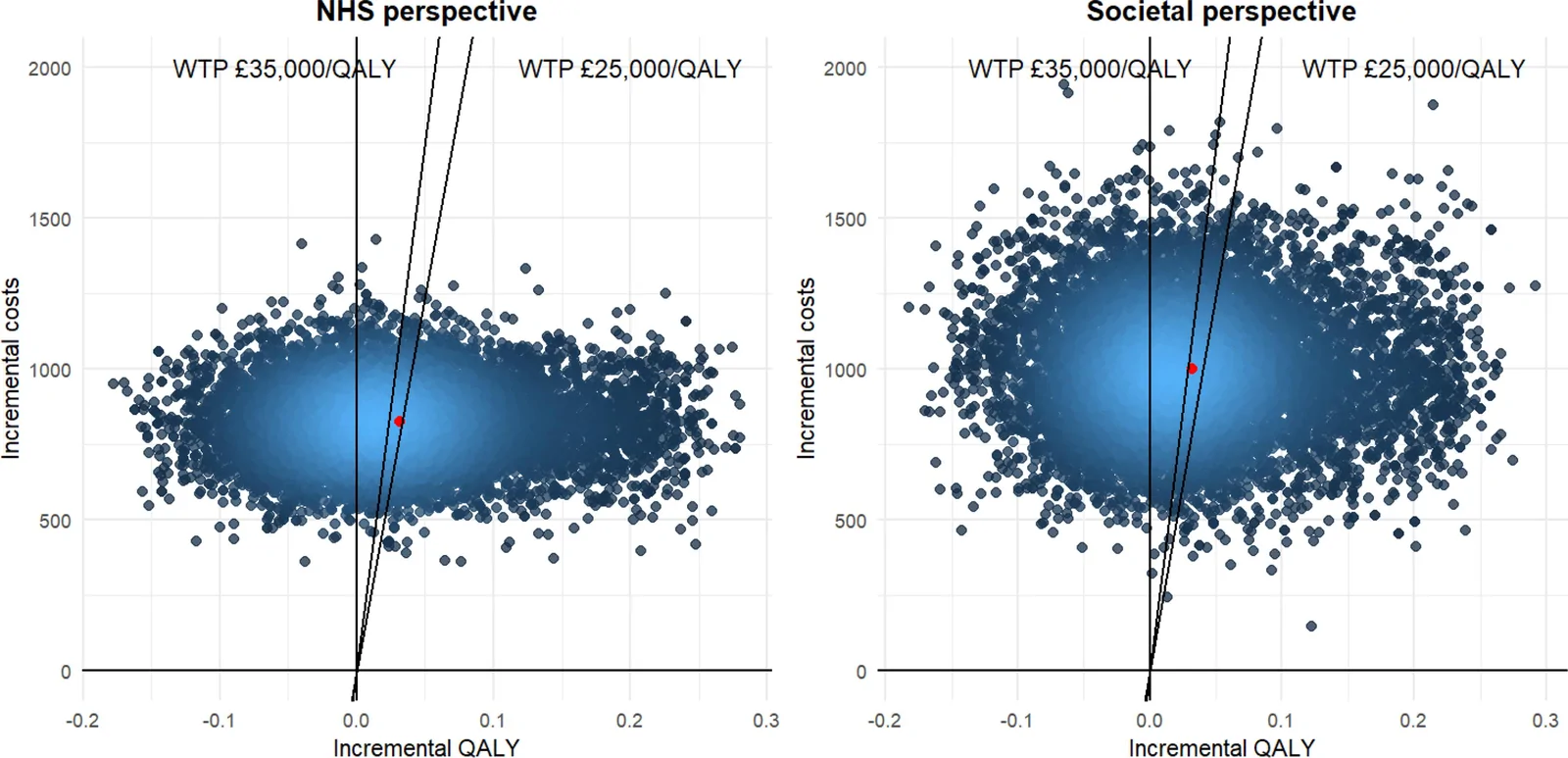
**Cost-effectiveness plane showing incremental costs and QALYs from Monte Carlo simulation for NeuroSAFE-guided RARP versus standard RARP. Each point represents one simulation draw. Diagonal lines indicate willingness-to-pay thresholds of £25,000 and £35,000 per QALY gained**.

**Fig. 2:**
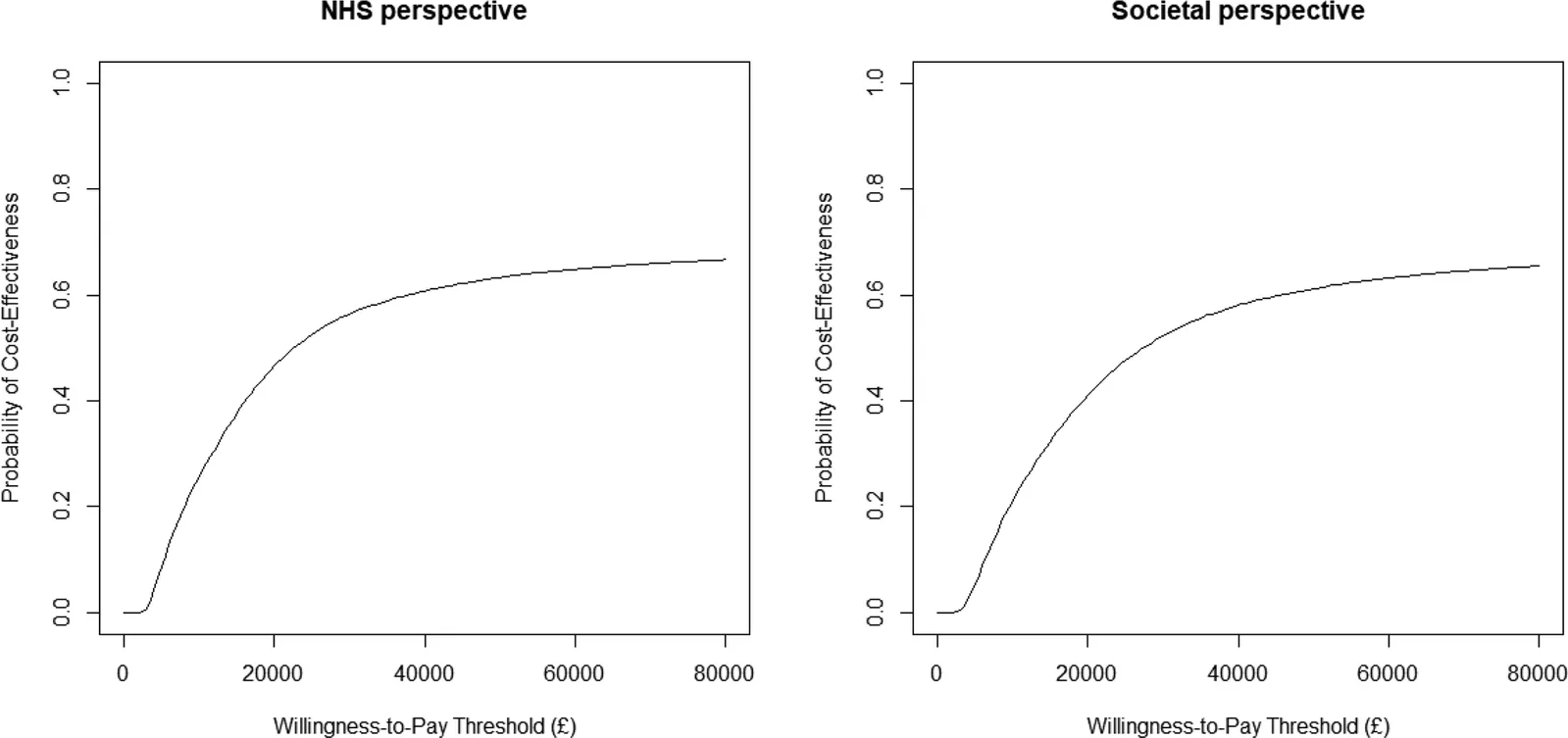
**Cost-effectiveness acceptability curves showing the probability that NeuroSAFE-guided RARP is cost-effective compared with standard RARP across willingness-to-pay thresholds, from NHS and societal perspectives**.

Figure 1 presents the simulation results for incremental QALYs and costs from the probabilistic sensitivity analysis. Most estimates lie in the upper right quadrant, indicating higher costs and utilities for NeuroSAFE-guided RARP, although a proportion of estimates fall in the upper-left quadrant, indicating higher costs with lower QALYs. The cost-effectiveness acceptability curves (Fig. 2) further demonstrate the probability of NeuroSAFE being cost-effective across different willingness-to-pay thresholds. The probabilities of being cost-effective were 53% and 56% (NHS perspective) and 48% and 56% (societal perspective) at the lower and upper NICE thresholds, respectively.

### 3.6 Interaction analysis

Table 5 presents the results of regressing NMBs on treatment assignment, subgroup indicator (patients not recommended for bilateral NS; 136 in standard RARP and 141 in NeuroSAFE-guided RARP), and their interaction term. The interaction terms showed positive contributions to NMBs among those receiving NeuroSAFE-guided RARP who were not recommended for NS. Nevertheless, these coefficients were not statistically significant. Mean costs, QALYs, ICERs, and NMBs for the subgroup are reported in Supplementary Table 7.

**Table 5:** Regression analysis of NMB on treatment and subgroup for patients not recommended for nerve-sparing

	**NHS perspective**				**Societal perspective**			
	**NMB at lower threshold**		**NMB at upper threshold**		**NMB at lower threshold**		**NMB at upper threshold**	
	**Model 1** **[95% CI]**	**Model 2** **[95% CI]**	**Model 1** **[95% CI]**	**Model 2** **[95% CI]**	**Model 1** **[95% CI]**	**Model 2** **[95% CI]**	**Model 1** **[95% CI]**	**Model 2** **[95% CI]**
Intercept	13 425 [10 281–16 569]	13 259 [10 103–16 415]	21 846 [17 433–26 258]	21 561 [17 147–25 976]	13 105 [9989–16 220]	13 032 [9 865–16 199]	21 525 [17 138–25 912]	21 334 [16 912–25 756]
Treatment	−38 [–4192, 4117]	−191 [–4748, 4365]	278 [–5551, 6107]	77 [–6299, 6453]	−211 [–4335, 3913]	−555 [–5124, 4013]	105 [–5695, 5906]	−287 [–6670, 6096]
Subgroup		245 [−1142, 1632]		420 [−1497, 2336]		107 [−1353, 1567]		281 [−1693, 2256]
Treatment × Subgroup		216 [−1900, 2333]		281 [−2650, 3211]		495 [−1715, 2706]		560 [−2445, 3565]

CI = confidence interval; NHS = National Health Service; NMB = net monetary benefit; QALY = quality-adjusted life year.

NMB at lower threshold: £25 000/QALY gained; NMB at upper threshold: £35 000/QALY gained.

Model 1: linear model of NMBs on treatment group; Model 2: linear model of NMBs on treatment group, subgroup for patients not recommended for nerve-sparing, and their interaction terms.

## 4 Discussion

The NeuroSAFE-PROOF trial demonstrated significant improvements in early return to continence and erectile function at 12 mo [6]. Our economic evaluation suggests that, compared with standard RARP, NeuroSAFE-guided RARP was associated with moderate additional NHS costs alongside incremental QALYs on average over the 12-mo period. From a societal perspective, results were less favourable, although the ICER was still within the upper NICE threshold. Cost differences were primarily driven by the additional resources required to perform the NeuroSAFE procedure, rather than the downstream health care utilisation. The observed QALY gain over 12 mo represents a modest average improvement in HRQoL. Although the trial demonstrated improvements in urinary continence and erectile function, these benefits may not have been fully captured by the EQ-5D-5L, a generic measure that may be relatively insensitive to PC-specific functional outcomes. Consequently, the QALY estimates may underestimate clinically meaningful benefits, particularly in the early postoperative period. The relatively small QALY differences make ICERs sensitive to variations in cost or utility values. This is reflected in the probabilities of cost effectiveness being around 50% at the NICE thresholds. Sensitivity analyses yielded consistent results.

According to our interaction analyses, point estimates suggested that NeuroSAFE offers potentially greater economic value for the subgroup not recommended for bilateral NS, compared with other patients. However, these findings should be interpreted cautiously, as the interaction analyses are exploratory, potentially underpowered, and associated with greater statistical uncertainty.

To our knowledge, this is the first cost-utility analysis of NeuroSAFE conducted within a large multicentre RCT in the UK. A key strength is the use of detailed patient-level data on resource utilisation and utility measured at multiple time points, across multiple economic perspectives.

Our study has several limitations. First, resource use data were collected retrospectively, potentially introducing recall bias; however, such bias is unlikely to differ systematically between NeuroSAFE and RARP groups. Second, due to incomplete details regarding reasons for NHS service utilisation, average unit costs for A&E visits, hospital admissions, and outpatient attendances were applied. Differences between groups in the actual reasons for health care use might have influenced cost estimations, although this potential bias was mitigated through extensive probabilistic sensitivity analysis. Although follow-up visits related to biochemical recurrence were included in the overall health care utilisation costs, the relatively small number of recurrence events and short follow-up period limit the ability to fully assess differences in longer-term management and associated costs. Third, incomplete cases comprised approximately 50% of the sample. To address this issue, we applied multiple imputation in accordance with recommended methodological guidelines for trial-based cost-effectiveness analysis [20]. Fourth, although the sample size is appropriate for the clinical trial, it may be limited for economic evaluation given the variability in cost data, and the study may be underpowered to detect small differences in costs and QALYs. Fifth, the estimated incremental operating theatre time associated with NeuroSAFE in this study reflects the average difference observed during the trial. In other settings, integration of the frozen section process into surgical workflow, such as performing pelvic lymph node dissection during the waiting period for pathology results, may reduce additional operating time. In such scenarios, the incremental cost of the NeuroSAFE procedure could potentially be lower than estimated in this analysis. Sixth, participants were informed of their NS status, which may have influenced patient-reported EQ-5D and CSRI outcomes. Knowledge of receiving NS surgery could lead to more favourable perceptions of recovery and lower reported health care utilisation, potentially biasing QALYs upward and costs downward in the NeuroSAFE group. This potential reporting bias should be considered when interpreting the results. Finally, our analysis employed a 12-mo time horizon aligned with the primary follow-up of the NeuroSAFE-PROOF trial. The absence of longer-term follow-up also limits the ability to extrapolate costs and outcomes beyond the trial period, and therefore the results should not be interpreted as reflecting lifetime cost effectiveness. These findings should be interpreted in the context of the trial population, which included men with good baseline erectile function.

Future studies incorporating longer follow-up could provide a more comprehensive assessment of the long-term economic impact of NeuroSAFE.

## 5 Conclusions

NeuroSAFE-guided RARP increases costs and generates modest QALY gains compared with standard RARP on average. ICERs were within commonly used UK willingness-to-pay thresholds, particularly the upper threshold (£35 000 per QALY); however, cost effectiveness remained uncertain, with probabilities below 60% and substantial variability in the estimates. These findings reflect short-term (12-mo) within-trial results and should not be interpreted as evidence of long-term cost effectiveness.

  ***Author contributions***: Elena Pizzo had full access to all the data in the study and takes responsibility for the integrity of the data and the accuracy of the data analysis.

  *Study concept and design*: Pizzo, Wang, Shaw, Dinneen.

*Acquisition of data*: Pizzo, Wang, Almeida-Magana.

*Analysis and interpretation of data*: Pizzo, Wang.

*Drafting of the manuscript*: Pizzo, Wang.

*Critical revision of the manuscript for important intellectual content*: Pizzo, Shaw, Dinneen, Almeida-Magana, Leurent.

*Statistical analysis*: Wang.

*Obtaining funding*: Shaw, Dinneen.

*Administrative, technical, or material support*: Brew-Graves, Roberts.

*Supervision*: Pizzo, Shaw.

*Other* (review, editing): Pizzo, Wang, Shaw, Dinneen, Haider, Almeida-Magana, Williams, Pen, Laurent.

  ***Financial disclosures:*** Elena Pizzo certifies that all conflicts of interest, including specific financial interests and relationships and affiliations relevant to the subject matter or materials discussed in the manuscript (eg, employment/affiliation, grants or funding, consultancies, honoraria, stock ownership or options, expert testimony, royalties, or patents filed, received, or pending), are the following: None.

  ***Funding/Support and role of the sponsor:*** The sponsor of this study was University College London, funders were the Rosetrees Foundation, the National Institute for Healthcare Research (NIHR) Research for Patient Benefit (RfPB) stream (reference: PB-PG-1216-200113), St Peter’s Charitable Trust, and the 10.13039/501100023262Jon Moulton Charity Trust (charity no. 1109891). JW and EP are supported by the 10.13039/501100000272National Institute for Health and Care Research ARC North Thames. No funding body had, nor will have any role in the design, conduct, analysis, interpretation, or dissemination of the study protocol or its final outputs. The views expressed are those of the author(s) and not necessarily those of the NIHR or the Department of Health and Social Care or any other organisations involved. The study/project is also supported by the NIHR Clinical Research Network (CPMS 50730).

  ***Data access and responsibility:*** EP had full access to all the data in the study and takes responsibility for the integrity of the data and the accuracy of the data analysis.

  ***Data sharing:*** Data are available for bona fide researchers who request it from the authors.

  ***Ethics approval and consent to participate:*** Ethical approval was obtained on 16/06/2021 with the North London REC, REC ref: 17/LO/1978. Written informed consent to participate was obtained from all participants before enrolment.

  ***Consent for publication:*** All model consent forms used in the trial are available on request.

  ***Declaration of generative AI and AI-assisted technologies in the writing process:*** The authors did not use generative artificial intelligence (AI) or AI-assisted technologies for the conception, analysis, interpretation, writing, or editing of this manuscript.

  ***Acknowledgements:*** This research would not have been possible without the support of the patients who took part, the research staff at each site, the National Cancer Imaging Translational Accelerator (NCITA), the clinical trials unit and the clinical teams who recruited participants and cared for the patients during the study.
