# Supplementary material for: Cost-utility of NeuroSAFE-guided RARP Versus Standard RARP in Men with Localised Prostate Cancer in the UK
Source: Eur Urol Open Sci. 2026 Sep 9;92:55–63. doi: 10.1016/j.euros.2026.08.007 (PMC13582013; doi:10.1016/j.euros.2026.08.007)
Supplement: Supplementary file 1 [file mmc1.docx]

**Table S1. Trajectory of costs for NeuroSAFE-guided RARP and Standard RARP (NHS and private), based on observed data.**

|  | **3 months** | | **6 months** | | **12 months** | |
| --- | --- | --- | --- | --- | --- | --- |
|  | **Standard RARP**  **(n = 110; n/N = 54%)** | **NeuroSAFE-guided RARP**  **(n = 113; n/N = 55%)** | **Standard RARP**  **(n = 117; n/N = 58%)** | **NeuroSAFE-guided RARP**  **(n = 113; n/N = 55%)** | **Standard RARP**  **(n = 117; n/N = 58%)** | **NeuroSAFE-guided RARP**  **(n = 137; n/N = 67%)** |
|  | **Cost (£) Mean (SD)** | **Cost (£) Mean (SD)** | **Cost (£) Mean (SD)** | **Cost (£) Mean (SD)** | **Cost (£) Mean (SD)** | **Cost (£) Mean (SD)** |
| **Medication** |  |  |  |  |  |  |
| NHS | 0.31 (0.83) | 0.99 (3.18) | 1.21 (4.34) | 1.09 (2.73) | 1.21 (4.16) | 1.66 (6.76) |
| Private | 1.81 (8.61) | 0.27 (1.87) | 2.61 (18.95) | 0.57 (5.26) | 1.06 (5.93) | 0.71 (3.75) |
| Total | 2.12 (8.59) | 1.26 (3.61) | 3.81 (19.35) | 1.67 (5.82) | 2.28 (7.06) | 2.37 (7.63) |
| **Device** |  |  |  |  |  |  |
| NHS | 53.92 (122.44) | 55.42 (120.06) | 81.65 (137.70) | 73.64 (132.95) | 86.76 (140.30) | 84.66 (139.05) |
| Private | 2.05 (13.99) | 1.48 (20.31) | 4.35 (33.93) | 7.20 (44.97) | 2.49 (22.68) | 3.08 (25.75) |
| Total | 55.97 (123.10) | 56.90 (121.09) | 86.00 (139.27) | 80.84 (136.50) | 89.25 (140.83) | 87.74 (139.67) |
| **Further cancer treatment** |  |  |  |  |  |  |
| NHS | 0.00 (0.00) | 0.63 (9.06) | 1.27 (18.16) | 24.24 (299.64) | 0.64 (9.08) | 47.63 (345.51) |
| Private | 0.00 (0.00) | 0.00 (0.00) | 0.00 (0.00) | 0.00 (0.00) | 0.00 (0.00) | 0.00 (0.00) |
| Total | 0.00 (0.00) | 0.63 (9.06) | 1.27 (18.16) | 24.24 (299.64) | 0.64 (9.08) | 47.63 (345.51) |
| **Accident & Emergency** |  |  |  |  |  |  |
| NHS | 14.25 (83.59) | 21.92 (81.69) | 6.48 (61.03) | 7.74 (51.63) | 6.48 (48.53) | 14.18 (74.77) |
| Private | 1.30 (18.46) | 0.00 (0.00) | 0.00 (0.00) | 0.00 (0.00) | 1.30 (18.46) | 0.00 (0.00) |
| Total | 15.55 (96.67) | 21.92 (81.69) | 6.48 (61.03) | 7.74 (51.63) | 7.77 (63.63) | 14.18 (74.77) |
| **Ambulance Call** |  |  |  |  |  |  |
| NHS | 0.06 (0.89) | 0.37 (3.05) | 0.37 (3.06) | 0.00 (0.00) | 0.00 (0.00) | 0.37 (4.51) |
| Private | 0.00 (0.00) | 0.00 (0.00) | 0.19 (1.98) | 0.00 (0.00) | 0.00 (0.00) | 0.00 (0.00) |
| Total | 0.06 (0.89) | 0.37 (3.05) | 0.56 (4.76) | 0.00 (0.00) | 0.00 (0.00) | 0.37 (4.51) |
| **Paramedics** |  |  |  |  |  |  |
| NHS | 2.40 (34.25) | 0.00 (0.00) | 0.00 (0.00) | 0.00 (0.00) | 0.00 (0.00) | 3.59 (38.12) |
| Private | 0.00 (0.00) | 0.00 (0.00) | 0.00 (0.00) | 0.00 (0.00) | 0.00 (0.00) | 0.00 (0.00) |
| Total | 2.40 (34.25) | 0.00 (0.00) | 0.00 (0.00) | 0.00 (0.00) | 0.00 (0.00) | 3.59 (38.12) |
| **Walk-in Centre** |  |  |  |  |  |  |
| NHS | 0.39 (5.61) | 3.92 (42.58) | 4.33 (26.64) | 2.35 (20.88) | 2.36 (15.74) | 1.57 (17.69) |
| Private | 0.00 (0.00) | 0.78 (11.20) | 0.00 (0.00) | 0.00 (0.00) | 0.00 (0.00) | 0.00 (0.00) |
| Total | 0.39 (5.61) | 4.71 (48.07) | 4.33 (26.64) | 2.35 (20.88) | 2.36 (15.74) | 1.57 (17.69) |
| **Hospital Inpatient** |  |  |  |  |  |  |
| NHS | 2.38 (23.96) | 9.49 (67.27) | 8.34 (84.72) | 2.37 (33.89) | 10.73 (73.44) | 27.28 (260.05) |
| Private | 0.00 (0.00) | 3.56 (50.83) | 0.00 (0.00) | 0.00 (0.00) | 0.00 (0.00) | 0.00 (0.00) |
| Total | 2.38 (23.96) | 13.05 (110.61) | 8.34 (84.72) | 2.37 (33.89) | 10.73 (73.44) | 27.28 (260.05) |
| **Hospital Outpatient** |  |  |  |  |  |  |
| NHS | 39.06 (113.29) | 46.65 (147.86) | 37.26 (152.36) | 34.69 (105.40) | 45.07 (159.51) | 39.47 (121.29) |
| Private | 2.40 (20.89) | 3.59 (38.12) | 1.20 (12.08) | 0.00 (0.00) | 2.40 (27.04) | 0.60 (8.54) |
| Total | 41.47 (118.80) | 50.24 (166.35) | 38.46 (153.51) | 34.69 (105.40) | 47.48 (165.62) | 40.07 (123.19) |
| **Day Hospital** |  |  |  |  |  |  |
| NHS | 0.60 (8.56) | 1.20 (12.05) | 1.20 (17.13) | 1.79 (19.06) | 3.00 (22.51) | 0.60 (8.54) |
| Private | 0.00 (0.00) | 0.00 (0.00) | 0.00 (0.00) | 0.60 (8.54) | 0.60 (8.56) | 0.00 (0.00) |
| Total | 0.60 (8.56) | 1.20 (12.05) | 1.20 (17.13) | 2.39 (20.84) | 3.61 (26.90) | 0.60 (8.54) |
| **GP** |  |  |  |  |  |  |
| NHS | 18.46 (48.51) | 21.33 (60.17) | 21.16 (66.25) | 17.92 (65.20) | 20.76 (69.68) | 22.49 (65.00) |
| Private | 2.48 (14.23) | 3.32 (23.19) | 2.34 (19.05) | 1.10 (9.49) | 4.25 (27.00) | 2.04 (14.51) |
| Total | 20.94 (54.61) | 24.65 (70.36) | 23.50 (69.43) | 19.02 (66.37) | 25.01 (93.36) | 24.53 (69.57) |
| **Physiotherapy** |  |  |  |  |  |  |
| NHS | 6.30 (75.50) | 1.83 (11.06) | 1.57 (14.26) | 3.65 (28.50) | 3.85 (29.30) | 3.13 (29.31) |
| Private | 0.76 (6.26) | 2.52 (30.43) | 0.00 (0.00) | 0.86 (7.32) | 0.27 (3.86) | 1.68 (10.26) |
| Total | 7.06 (75.69) | 4.35 (32.23) | 1.57 (14.26) | 4.51 (29.32) | 4.12 (29.52) | 4.81 (30.88) |
| **Other specialist** |  |  |  |  |  |  |
| NHS | 22.11 (218.63) | 9.88 (76.12) | 4.63 (32.72) | 2.95 (18.31) | 6.35 (40.39) | 9.26 (46.02) |
| Private | 0.30 (4.28) | 0.00 (0.00) | 0.60 (8.56) | 0.99 (10.67) | 3.57 (36.76) | 1.81 (21.34) |
| Total | 22.41 (218.81) | 9.88 (76.12) | 5.23 (33.74) | 3.03 (21.06) | 9.92 (73.07) | 11.07 (59.70) |
| **Other Equipment** |  |  |  |  |  |  |
| NHS | 0.00 (0.00) | 0.00 (0.00) | 0.00 (0.00) | 0.00 (0.00) | 0.00 (0.00) | 0.00 (0.00) |
| Private | 11.24 (31.21) | 14.61 (63.76) | 4.70 (18.44) | 4.83 (24.74) | 9.79 (44.59) | 19.77 (130.47) |
| Total | 11.24 (31.21) | 14.61 (63.76) | 4.70 (18.44) | 4.83 (24.74) | 9.79 (44.59) | 19.77 (130.47) |
| **Productivity Loss** | 82.20 (260.68) | 76.00 (257.84) | 28.90 (129.96) | 25.40 (104.48) | 31.24 (163.68) | 36.92 (231.50) |
| **Family and paid carer** | 59.38 (258.67) | 82.83 (342.64) | 23.99 (211.07) | 84.08 (520.43) | 14.24 (78.22) | 98.42 (793.82) |
| **Travel** | 27.10 (105.44) | 10.65 (30.25) | 6.95 (24.82) | 10.93 (53.47) | 15.58 (62.63) | 14.25 (61.11) |

*The calculations were based on trial data collected through the Client Service Receipt Inventory (CSRI) questionnaire, combined with unit costs derived from National Schedule of NHS costs, PSSRU 2023/4 and British National Formulary (BNF) 2023/4.*
