# Supplementary material for: Cost-utility of NeuroSAFE-guided RARP Versus Standard RARP in Men with Localised Prostate Cancer in the UK
Source: Eur Urol Open Sci. 2026 Sep 9;92:55–63. doi: 10.1016/j.euros.2026.08.007 (PMC13582013; doi:10.1016/j.euros.2026.08.007)
Supplement: Supplementary file 2 [file mmc2.docx]

**Table S2. Complete-case analysis without imputation: mean costs, QALYs, ICER and NMBs (NHS and societal perspectives)**

|  | **NHS perspective** | | **Societal perspective** | |
| --- | --- | --- | --- | --- |
|  | **Standard RARP** | **NeuroSAFE-guided RARP** | **Standard RARP** | **NeuroSAFE-guided RARP** |
| Intervention cost | £7,076 | £7,829 | £7,076 | £7,829 |
| Health service cost | £523 | £595 | £696 | £900 |
| Productivity loss | N/A | N/A | £138 | £130 |
| Total cost | £7,599 | £8,424 | £7,909 | £8,859 |
| Incremental cost [95% CI] |  | £825 [£656 to £994] |  | £950 [£666 to £1253] |
| QALY | 0.86 | 0.89 | 0.86 | 0.89 |
| Incremental QALY [95% CI] |  | 0.03 [0.00 to 0.07] |  | 0.03 [0.00 to 0.07] |
| ICER (extra cost per QALY gained) |  | £24,319 |  | £27,997 |
| Net monetary benefit (CE threshold: 25k) | £13,904 | £13,927 | £13,593 | £13,492 |
| Net monetary benefit (CE threshold: 35k) | £22,505 | £22,867 | £22,194 | £22,432 |
| Incremental NMB (CE threshold: 25k) |  | £23 |  | -£102 |
| Incremental NMB (CE threshold: 35k) |  | £362 |  | £238 |

Analyses were based on patients with complete cost and utility data (RARP: n = 112/203; NeuroSAFE-guided RARP: n = 99/204). Confidence intervals were derived from non-
parametric bootstrap using the percentile method.
