# Supplementary material for: Cost-utility of NeuroSAFE-guided RARP Versus Standard RARP in Men with Localised Prostate Cancer in the UK
Source: Eur Urol Open Sci. 2026 Sep 9;92:55–63. doi: 10.1016/j.euros.2026.08.007 (PMC13582013; doi:10.1016/j.euros.2026.08.007)
Supplement: Supplementary file 3 [file mmc3.docx]

**Table S3. Sensitivity analyses by removing patients received adjuvant radiation therapy, without imputation: mean costs, QALYs, ICER and NMBs (NHS and societal perspectives)**

|  | **NHS perspective** | | **Societal perspective** | |
| --- | --- | --- | --- | --- |
|  | **Standard RARP** | **NeuroSAFE-guided RARP** | **Standard RARP** | **NeuroSAFE-guided RARP** |
| Intervention cost | £7,076 | £7,829 | £7,076 | £7,829 |
| Health service cost | £521 | £596 | £695 | £908 |
| Productivity loss | N/A | N/A | £139 | £121 |
| Total cost | £7,597 | £8,425 | £7,910 | £8,858 |
| Incremental cost [95% CI] |  | £829 [£660 to £1019] |  | £948 [£655 to £1284] |
| QALY | 0.86 | 0.90 | 0.86 | 0.90 |
| Incremental QALY [95% CI] |  | 0.04 [0.00 to 0.07] |  | 0.04 [0.00 to 0.07] |
| ICER (extra cost per QALY gained) |  | £23,590 |  | £26,982 |
| Net monetary benefit (CE threshold: 25k) | £13,906 | £13,956 | £13,593 | £13,523 |
| Net monetary benefit (CE threshold: 35k) | £22,507 | £22,908 | £22,194 | £22,475 |
| Incremental NMB (CE threshold: 25k) |  | £50 |  | -£70 |
| Incremental NMB (CE threshold: 35k) |  | £401 |  | £282 |

Ten patients received adjuvant radiation therapy, which is no longer standard of care due to changes in evidence since the start of the study (RARP: n = 2; NeuroSAFE-guided RARP: n = 8). In this sensitivity analysis, we analysed data for patients not receiving adjuvant radiation therapy and with complete cost and utility data (RARP: n = 112/203; NeuroSAFE-guided RARP: n = 94/204). Confidence intervals were derived from non-parametric bootstrap using the percentile method.
