# Supplementary material for: Cost-utility of NeuroSAFE-guided RARP Versus Standard RARP in Men with Localised Prostate Cancer in the UK
Source: Eur Urol Open Sci. 2026 Sep 9;92:55–63. doi: 10.1016/j.euros.2026.08.007 (PMC13582013; doi:10.1016/j.euros.2026.08.007)
Supplement: Supplementary file 4 [file mmc4.docx]

**Table S4. Sensitivity analyses for the departure from the missing-at-random assumption**

| **Scenario description** | **Incremental cost (£) NHS perspective  [95% CI]** | **Incremental cost (£) Societal perspective  [95% CI]** | **Incremental QALY [95% CI]** | **INMB at lower threshold (£) NHS perspective [95% CI]** | **INMB at upper threshold (£) NHS perspective [95% CI]** | **INMB at lower threshold (£) Societal perspective [95% CI]** | **INMB at upper threshold (£) Societal perspective [95% CI]** | **Bootstrap cost-effectiveness probability at lower threshold NHS perspective** | **Bootstrap cost-effectiveness probability at upper threshold NHS perspective** | **Bootstrap cost-effectiveness probability at lower threshold Societal perspective** | **Bootstrap cost-effectiveness probability at upper threshold Societal perspective** |
| --- | --- | --- | --- | --- | --- | --- | --- | --- | --- | --- | --- |
| MAR | 827 [649 to 1007] | 1000 [694 to 1338] | 0.03 [-0.01 to 0.21] | -38 [-3249 to 4353] | 278 [-4215 to 6423] | -211 [-3429 to 4189] | 105 [-4390 to 6260] | 53% | 60% | 47% | 56% |
| MNAR: -10% QALY in both arms | 827 [649 to 1007] | 1000 [694 to 1338] | 0.03 [-0.13 to 0.20] | -30 [-4145 to 4112] | 289 [-5469 to 6077] | -203 [-4318 to 3949] | 117 [-5644 to 5920] | 46% | 55% | 41% | 50% |
| MNAR: +10% cost in both arms | 848 [680 to 1020] | 1028 [732 to 1361] | 0.03 [-0.13 to 0.20] | 151 [-4412 to 4744] | 551 [-5834 to 6980] | -28 [-4586 to 4574] | 371 [-6008 to 6810] | 52% | 60% | 46% | 56% |
| MNAR: -10% QALY and + 10% cost | 848 [680 to 1020] | 1028 [732 to 1361] | 0.03 [-0.13 to 0.20] | -51 [-4167 to 4083] | 269 [-5490 to 6060] | -230 [-4348 to 3922] | 89 [-5681 to 5892] | 46% | 54% | 40% | 50% |
| MNAR: -10% QALY in the NeuroSAFE arm | 827 [649 to 1007] | 1000 [694 to 1338] | 0.00 [-0.18 to 0.17] | -920 [-5219 to 3487] | -956 [-6971 to 5216] | -1092 [-5384 to 3323] | -1129 [-7137 to 5049] | 27% | 30% | 25% | 28% |
| MNAR: -10% QALY in control arm | 827 [649 to 1007] | 1000 [694 to 1338] | 0.08 [-0.10 to 0.25] | 1062 [-3317 to 5390] | 1817 [-4316 to 7874] | 889 [-3488 to 5219] | 1644 [-4476 to 7703] | 75% | 79% | 71% | 77% |
| MNAR: +10% cost in the NeuroSAFE arm | 1185 [1016 to 1357] | 1370 [1068 to 1707] | 0.03 [-0.01 to 0.21] | -186 [-4747 to 4409] | 214 [-6171 to 6643] | -371 [-4928 to 4231] | 29 [-6352 to 6466] | 41% | 52% | 37% | 48% |
| MNAR: +10% cost in the control arm | 490 [316 to 471] | 658 [357 to 995] | 0.03 [-0.01 to 0.21] | 509 [-4053 to 5104] | 909 [-5476 to 7340] | 342 [-4219 to 4943] | 741 [-5635 to 7181] | 62% | 66% | 58% | 63% |

INMB: incremental net monetary benefit; MAR: missing at random; MNAR: missing not at random

We applied percentage changes to imputed values in eight different scenarios: 1) base-case results where data were assumed to be missing at random; 2) missing QALYs were 10% lower than the imputed values in both arms; 3) missing costs were 10% higher than the imputed values in both arms; 4) missing QALYs were 10% lower and missing costs were 10% higher than the imputed values in both arms; 5) missing QALYs were 10% lower than the imputed values in the NeuroSAFE arm; 6) missing QALYs were 10% lower than the imputed values in the control arm; 7) missing costs were 10% higher than the imputed values in the NeuroSAFE arm; 8) missing costs were 10% than the imputed values in the control arm.

It should be noted that, in the main text, we presented cost-effectiveness probabilities derived from the probabilistic sensitivity analysis. On the other hand, the cost-effectiveness probabilities presented here were obtained from the 1,000 bootstrap replicates. Therefore, the cost-effectiveness probabilities under the MAR scenario presented here are not identical to those reported in the main text.
