# Supplementary material for: Cost-utility of NeuroSAFE-guided RARP Versus Standard RARP in Men with Localised Prostate Cancer in the UK
Source: Eur Urol Open Sci. 2026 Sep 9;92:55–63. doi: 10.1016/j.euros.2026.08.007 (PMC13582013; doi:10.1016/j.euros.2026.08.007)
Supplement: Supplementary file 5 [file mmc5.docx]

**Table S5. Probabilistic sensitivity results: mean costs, QALYs, ICER and NMBs (NHS perspective)**

|  | **Standard RARP** | **NeuroSAFE-guided RARP** |
| --- | --- | --- |
| Intervention cost | £7,076 | £7,829 |
| Health service cost | £550 | £624 |
| Total cost | £7,626 | £8,453 |
| Incremental cost [95% CI] |  | £827 [£574 to £1088] |
| QALY | 0.84 | 0.87 |
| Incremental QALY [95% CI] |  | 0.03 [-0.01 to 0.21] |
| ICER (extra cost per QALY gained) |  | £26,113 |
| Net monetary benefit (CE threshold: 25k) | £13,422 | £13,387 |
| Net monetary benefit (CE threshold: 35k) | £21,841 | £22,123 |
| Incremental NMB (CE threshold: 25k) |  | -£35 |
| Incremental NMB (CE threshold: 35k) |  | £282 |

Values represent mean estimates from probabilistic simulations pooled across imputed datasets using Rubin’s rules.

Confidence intervals were derived from non-parametric bootstrap using the percentile method.
