# Supplementary material for: Cost-utility of NeuroSAFE-guided RARP Versus Standard RARP in Men with Localised Prostate Cancer in the UK
Source: Eur Urol Open Sci. 2026 Sep 9;92:55–63. doi: 10.1016/j.euros.2026.08.007 (PMC13582013; doi:10.1016/j.euros.2026.08.007)
Supplement: Supplementary file 7 [file mmc7.docx]

**Table S7. Subgroup analysis for patients not recommended for bilateral nerve-sparing following multiple imputation: mean costs, QALYs, ICER and NMBs (NHS and societal perspectives)**

|  | **NHS perspective** | | **Societal perspective** | |
| --- | --- | --- | --- | --- |
|  | **Standard RARP** | **NeuroSAFE-guided RARP** | **Standard RARP** | **NeuroSAFE-guided RARP** |
| Intervention cost | £7,076 | £7,829 | £7,076 | £7,829 |
| Health service cost | £612 | £666 | £808 | £997 |
| Productivity loss | N/A | N/A | £169 | £119 |
| Total cost | £7,688 | £8,495 | £8,053 | £8,945 |
| Incremental cost [95% CI] |  | £807 [£585 to £1024] |  | £892 [£554 to £1249] |
| QALY | 0.85 | 0.88 | 0.85 | 0.88 |
| Incremental QALY [95% CI] |  | 0.03 [-0.07 to 0.21] |  | 0.03 [-0.07 to 0.21] |
| ICER (extra cost per QALY gained) |  | £24,250 |  | £26,805 |
| Net monetary benefit (CE threshold: 25k) | £13,503 | £13,529 | £13,138 | £13,079 |
| Net monetary benefit (CE threshold: 35k) | £21,979 | £22,339 | £21,614 | £21,888 |
| Incremental NMB (CE threshold: 25k) |  | £26 |  | -£59 |
| Incremental NMB (CE threshold: 35k) |  | £359 |  | £274 |

Values represent mean estimates from regression analyses pooled across imputed datasets using Rubin’s
 rules. Confidence intervals were derived from non-parametric bootstrap using the percentile method.
